# Growth and wetting of water droplet condensed between micron-sized particles and substrate

**DOI:** 10.1038/srep30989

**Published:** 2016-08-04

**Authors:** Tran Si Bui Quang, Fong Yew Leong, Hongjie An, Beng Hau Tan, Claus-Dieter Ohl

**Affiliations:** 1 A*STAR Institute of High Performance Computing, 1 Fusionopolis Way, Connexis, 138632, Singapore; 2 Division of Physics and Applied Physics, School of Physical and Mathematical Sciences, Nanyang Technological University, 21 Nanyang Link, 637371, Singapore

## Abstract

We study heterogeneous condensation growth of water droplets on micron-sized particles resting on a level substrate. Through numerical simulations on equilibrium droplet profiles, we find multiple wetting states towards complete wetting of the particle. Specifically, a partially wetting droplet could undergo a spontaneous transition to complete wetting during condensation growth, for contact angles above a threshold minimum. In addition, we find a competitive wetting behavior between the particle and the substrate, and interestingly, a reversal of the wetting dependence on contact angles during late stages of droplet growth. Using quasi-steady assumption, we simulate a growing droplet under a constant condensation flux, and the results are in good agreement with our experimental observations. As a geometric approximation for particle clusters, we propose and validate a pancake model, and with it, show that a particle cluster has greater wetting tendency compared to a single particle. Together, our results indicate a strong interplay between contact angle, capillarity and geometry during condensation growth.

Condensation often occurs when water vapor undergoes physical change in state on a solid surface^1^. This subject has many applications in many fields such as thin film growth^2^, heat transfer^3^, recovery of atmospheric water^4^,^5^ and polymer templating^6^. Relevant experimental and theoretical works can be found in the review paper of Ucar *et al*.^7^.

Of particular importance and interest is the growth rate of water droplets on a flat substrate^7^. Beysen and co-workers^8^,^9^,^10^,^11^ observed a power-law growth rate of one-third and explained it using a diffusive growth mechanism, where adsorbed water monomers diffuse laterally towards the droplet perimeter. The one-third power law exponent is corroborated by other studies, including those of Briscoe and Galvin^12^, Ucar and Erbil^13^,^14^ and Ichikawa *et al*.^15^. In contrast, direct condensation of saturated water vapor onto the droplet surface leads to a power law growth exponent of one-half^16^,^17^,^18^,^19^,^20^. The same one-half power law is experimentally observed for the droplet growth on hydrophobic substrate^21^,^22^. Still others found a linear relationship between droplet radius and time during condensation growth, due to an assumed constant water flux^23^,^24^,^25^. Together, this suggests that typical droplet growth exponent could range from one-third to unity.

A closely related phenomenon, capillary condensation, could occur at under-saturated pressures^26^ and find particular importance in porous media condensation^26^ and atomic force microscopy (AFM) measurement^27^,^28^,^29^. On a concave curvature meniscus, the saturation pressure on the meniscus reduces follows the Kelvin equation^30^. For capillary condensation on AFM tip, a thin layer of liquid atom is first absorbed on the flat substrate and particle surfaces, before coalescence occurs forming a liquid neck.

Generally, droplet growth is governed by transport mechanisms, namely, molecular diffusion, Knudsen diffusion, surface diffusion and thin film dynamics^27^,^28^,^29^. For molecular diffusion, the rate of growth of capillary condensates follows a limited diffusion model based on Langmuir theory of growth^31^. For Knudsen diffusion, the mean free path is much larger than the geometric gap size between the particle and flat substrate, so condensation rates are controlled by collisions between water vapor molecules and solid surfaces^27^,^29^. For surface diffusion, the accumulation of drop mass depends on the diffusivity of water monomers on the substrate^11^. Lastly, for thin film dynamics, thin adsorbed water film on the flat substrate flows towards the liquid neck, so condensation rates depends on the viscosity of the water liquid layer.

Here, we investigate the condensation growth of a water droplet on spherical solid particles on a flat substrate. To that, we develop and present a numerical model based on capillary pressure, contact angle and droplet morphology, and compare results against our experimental observations. In particular, we distinguish the behavior of droplet on a single spherical particle with that of a monolayer cluster on a flat substrate.

## Methods

### Theory

**Figure 1:**
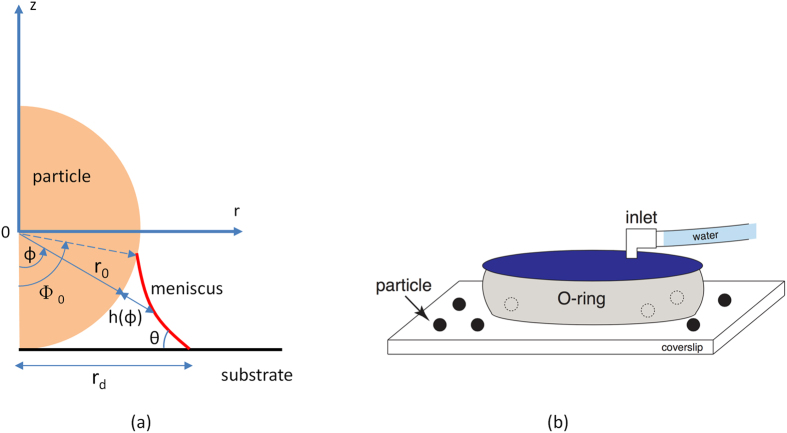
Schematic diagram and experimental set-up. (**a**) Schematic diagram of a water droplet attached to a spherical particle resting on horizontal substrate in axisymmetric spherical coordinates. (**b**) Experimental set-up.

Consider a liquid meniscus adhering to a spherical particle on a flat surface as shown in Fig. 1a. Using polar coordinates (r, ϕ, z), we define r_0_ as the radius of the particle, *ϕ* ∈ {0, 180°} as the polar angle taken from the negative direction (−z) on the vertical axis, Φ_0_ as the polar angle of the locus where the meniscus contacts the particle (wetting edge), θ as the in-plane tangential meniscus contact angle, h(ϕ) as the meniscus depth taken from the particle surface and r_d_ is the droplet-substrate contact radius taken from the axis of symmetry.

The droplet shape profile is governed by the Young-Laplace equation^32^,


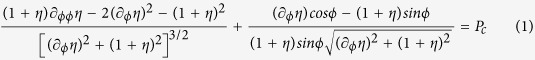


where *P*_*C*_ = *r*_*0*_*p*_*c*_/*σ* is the normalized capillary pressure, σ is the surface tension, p_c_ is the capillary pressure, *η*(*ϕ*) = *h*(*ϕ*)/*r*_*0*_ is the normalized meniscus thickness and 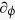 denotes the spatial derivative in polar coordinates. In Eq. (1), the terms on the left-hand side represent the in-plane and out-plane curvatures of the meniscus respectively, and the right-hand side the capillary pressure effect.

Based on our experimental study (detailed later), the droplet radius grows almost proportionally in time. The observed linear growth rate suggests a mechanism whereby water vapor condenses directly on the liquid meniscus during droplet growth^23^,^24^,^25^.

Following mass conservation, the droplet volume evolves as


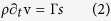


where Γ is the direct condensation mass flux, s is the meniscus surface area, v is the droplet volume, ρ is liquid density and t is time. For convenience, we can also express Eq. (2) in dimensionless form as 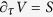, where *V* *=* *v*/*r*_0_^3^ is the normalized droplet volume, *S* = *s/r*_*0*_^2^ is the normalized meniscus surface and 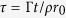 is the normalized condensation time.

The equilibrium meniscus droplet shape is obtained by solving Eq. (1) using an iterative shooting procedure implemented by a fourth order Runge-Kutta method. For a given meniscus contact point Φ_0_ (where η(Φ_0_) = 0), and surface contact angle θ (where 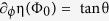), we vary the capillary pressure p_c_ until the resultant droplet profile intersects the flat substrate at the imposed contact angle of θ. At a given time, the resultant droplet profile is compared against the desired droplet volume obtained by Eq. (2), and the process is repeated iteratively until volume convergence is achieved.

The above numerical procedure assumes that thermodynamic equilibrium time-scale is fast compared to droplet growth time-scale, so that droplet growth is quasi-steady. Furthermore, we assume that both droplet-particle and droplet-substrate contact angles are of the same constant value, since both particle and substrate used in our experiments are made of silica.

### Experiment

We directly visualize water droplet growing on spherical particles via condensation, using an experimental setup shown in Fig. 1(b). To prepare the substrate, glass cover-slips (Menzel Glaser, Germany) were sonicated for 20 minutes each in acetone, isopropyl alcohol and ethanol and dried in a fast stream of nitrogen air. Separately, we determined the contact angle of a water droplet on the prepared glass slides to be approximately 22°. The particles were introduced via a stock solution of 1 μm silicon dioxide particles (1%, Thermo Scientific, United States), which was diluted by a factor of 10 in water before it was dispensed on the coverslip. The experiment was performed within a fluid cell (Bruker Corporation, United States) sealed by a silicone O-ring. Apart from inlet and outlet ports (closed during the experiment), the fluid cell is fully sealed at all times.

As a source of water vapor, liquid water was piped via silicone tubing up to the point just outside the inlet of the cell. We point out that no liquid water was delivered directly into the cell. The evaporating water creates a humid environment within the cell, which in turn promotes the condensation of water on the particles. We imaged the condensation and droplet growth from below the cover-slip via a 60x microscope objective (Olympus, Japan). The recording was performed with a CCD camera (Pixelfly QE, PCO AG, Germany) at a temporal resolution of 10 frames per second and a spatial resolution of 100 nm per pixel.

## Results

### Wetting transition

**Figure 2:**
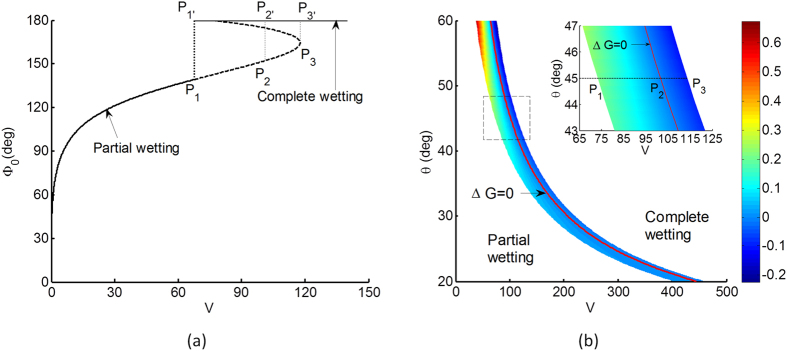
(**a**) Plot of wetting edge Φ_0_ on spherical particle against normalized droplet volume V, for a droplet contact angle of θ = 45°. Different wetting states are possible for a given droplet volume due to out-of-plane curvature effects. P_1_ indicates the onset of multiple wetting states, P_2_ the spontaneous transition from partial to complete wetting based on Gibbs free energy consideration and P_3_ the partial wetting limit. Here, with increasing droplet volume, continuous partial wetting occurs, until P_2_ where it jumps discontinuously to a complete wetting state at P_2′_ (**b**) Phase diagram of Gibbs energy difference ΔG between complete (upper right) and partial (lower left) wetting states, and the dependence on contact angles θ and droplet volumes V. The red contour depicts the transition curve ΔG = 0. The inset is a close-up on the dashed box region with the same parameter space used in (**a**).

First we consider the wetting of the particle and how it relates to the droplet volume and contact angle. For contact angle θ = 45°, we vary the wetting edge at the polar angle Φ_0_ from 30° to 180° (complete wetting) and numerically solve for droplet profiles against associated droplet volumes. Figure 2a shows that the drop volume increases monotonically with increasing wetting Φ_0_, until the turning point P_3_ is reached. Thereafter, the droplet volume at equilibrium apparently decrease with further wetting until complete wetting is reached at point P_1′_. This apparent reversal in wetting and volume trends can be attributed to a dramatic increase in out-plane curvature term in the Young-Laplace equation, which tends towards singularity as 1/r. This we corroborated using a separate analysis on droplet growth on an infinite 2D cylinder, and accordingly did not observe any wetting-volume reversal.

The dash section of the curve (Fig. 2a) from P_1_ to P_3_ represents multiple wetting states for the same droplet volume. Here the intermediate point P_2_ indicates spontaneous transition from partial wetting to complete wetting based on free energy considerations. The apparent wetting-volume reversal reflects a duality in wetting states, for instance the loci pair P_1_-P_1′_ and P_2_-P_2′_ represent partial and complete wetting states at the same droplet volume V (Fig. 2a). The thermodynamic consideration is that the droplet tends to adopt the wetting state which corresponds to a lower Gibbs free energy. Here, the Gibbs free energy G is expressed in dimensionless form as^33^,^34^,^35^


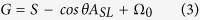


where G is scaled by σr_0_^2^, θ is the contact angle between droplet and flat surface and between droplet and spherical particle, *A*_*SL*_ is the scaled solid-liquid surface, S is the scaled water-air interface area and Ω_0_ is an constant.

Here we define ΔG as the difference in Gibbs energies between complete wetting (e.g. P_1′_ and P_3′_) and partial wetting (e.g. P_1_ and P_3_) states. Figure 2b shows a phase diagram of energy difference ΔG and its dependence on contact angle θ and droplet volume V. Here, we sketch the red contour curve for ΔG = 0, which separates distinct regions of positive (lower left) and negative (upper right) energy differences, and thus represents the threshold for complete wetting transition. Specifically, the inset shows a close-up of the dashed box region, and covers the parameter space used in Fig. 2a. The inset clearly shows that ΔG is positive between the loci range P_1_-P_1′_ to P_2_-P_2′_, but is negative between P_2_-P_2′_ to P_3_-P_3′_. This means that in the absence of wetting energy barriers, an initial partial wetting state could transit spontaneously to a complete wetting state with increasing droplet volume between P_1_ and P_3_.

### Capillary pressure and droplet shape

**Figure 3:**
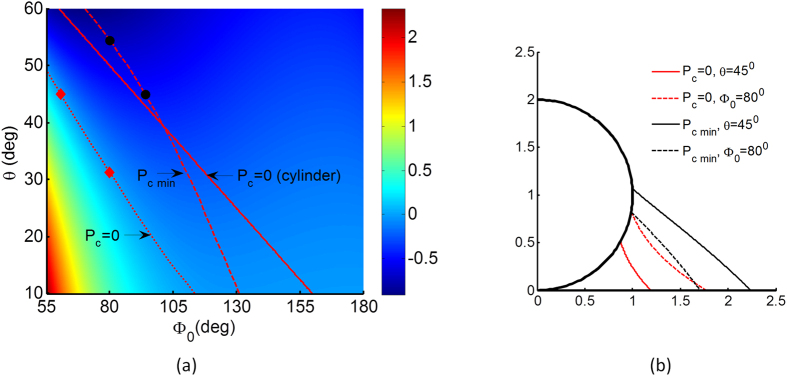
(**a**) The non-dimensional capillary pressure P_c_ of the droplet growth on the particle for various contact angles from 10° to 60°. (**b**) Droplet shape on the particle for contact angle θ = 45° and wetting edge Φ_0_ = 80° at zero capillary pressure P_c_ = 0 and at minimum non-dimensional capillary pressure P_c_ = P_c min_. The menisci are concave and convex for P_c_ = 0 and P_c_ = P_c min_ cases, respectively.

Next we turn to the droplet profile and its surface curvature at equilibrium. Figure 3a shows the phase diagram of the capillary pressure P_c_ as a function of the wetting edge Φ_0_ and contact angle θ. As indicated by the Young Laplace equation, the capillary pressure represents the sum of in-plane and out-of-plane surface curvature components. Due to the inherent centerline axisymmetry, the out-of-plane component is always negative, so the capillary pressure sums to zero if a positive in-plane curvature exactly cancels the out-of-plane component. The null capillary isobar P_c_ = 0 is sketched on Fig. 3a as red dotted curve, separating regions of surface concavity (lower left) and convexity (upper right). Here we define a minimum capillary isobar P_c,min_, which also represents the minimum in surface concavity, sketched as dashed curve. For comparison, we plot the null capillary isobar for the case of a 2D cylinder solved analytically as θ = (π−Φ_0_)/2, sketched here as red line.

Figure 3b shows in-plane droplet profiles held at either P_c_ = 0 or P_c,min_ for contact angle θ of 45° and wetting edge Φ_0_ of 80°. The corresponding parameter loci are indicated in Fig. 3a as red and black dot markers for reference. It is clear that the in-plane curvatures are consistently concave for null capillary pressure P_c_ = 0 and more linear for minimum capillary pressure P_c,min_.

**Figure 4:**
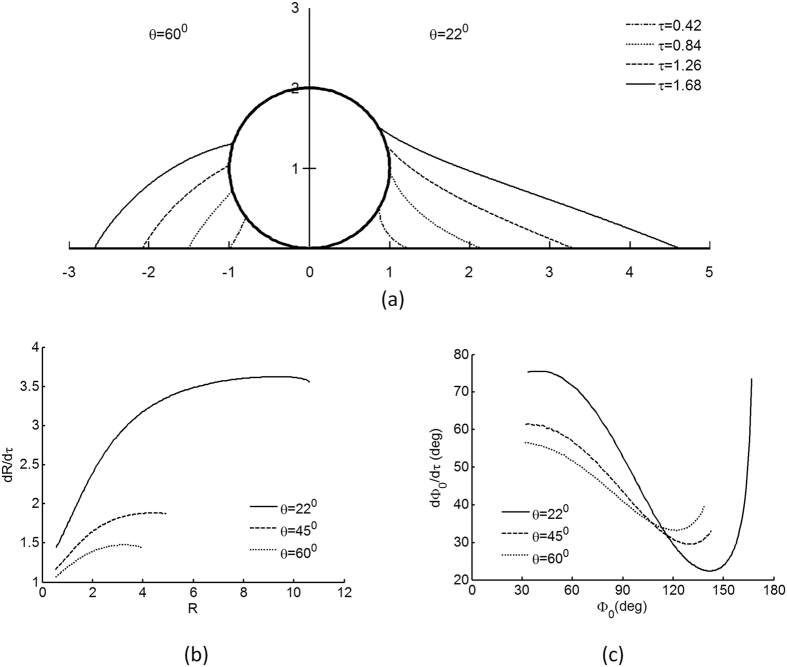
(**a**) Snapshots of droplet meniscus profiles taken in time increments of 0.42 τ, for contact angles of 22° on the right and 60° on the left side of the vertical aSxis. The initial volume of the droplet is V = 0.05 and the condensation rate is 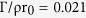 s^−1^. (**b**) The substrate wetting rate dR/dτ increases with droplet radius R at early times but tapers off later. (**c**) The particle wetting rate dΦ_0_/dτ decreases with wetting edge Φ_0_ at early times, but increases significantly as it approaches complete particle wetting. Both (**b**) and (**c**) are shown for contact angles θ = 22°, 45°, 60°.

Of further interest are the in-plane droplet growth profiles and its dependence on contact angles. Figure 4a shows the growth of in-plane droplet profiles on a single particle simulated using a condensation rate constant 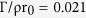 s^−1^ obtained from experiments (detailed later), in dimensionless time increments of 0.42 τ, and we compare the effects of contact angles θ of 60° (left) and 22° (right). Initially concave, the in-plane curvatures become increasingly convex with time, due to the effects of decreasing capillary pressure (Fig. 3). This evolution of in-plane curvatures is particularly evident at higher contact angles.

Figure 4b shows the droplet growth rate as a function of its instantaneous dimensionless radius R scaled by particle radius r_0_. Smaller droplets tend to spread laterally on the substrate slower compared to larger droplets, due to the competitive wetting of the particle at smaller droplet volumes. In addition, smaller contact angles correspond to faster spreading rates compared to larger contact angles, across all droplet sizes investigated. This is expected because a small contact angle is indicative of a hydrophilic surface, which tends to wet more easily compared to a hydrophobic one.

Figure 4c shows the rate of wetting as a function of its wetting edge Φ_0_. For a small wetting edge, the wetting rate decreases with wetting edge Φ_0_, as liquid accumulates preferentially in the bulk of the droplet due to curvature changes; the trend is however reversed towards complete wetting. As before, smaller contact angles θ (hydrophilic surface) lead to faster initial wetting rates, but this trend is reversed within a wetting interval of 120° and 150°, such that larger contact angles (hydrophobic surface) correspond to faster wetting rates.

The latter observation is, in particular, of interest, for it suggests an interaction between the contact angle, capillarity and the geometric constraint imposed by the spherical particle. Although the substrate and the particle are made of a common material with the same contact angle, the competition for surface wetting is unbalanced, and preferential wetting occurs depending on the specific drop volume, geometry and thermodynamics.

### Condensation growth rates

In our experiment, water vapor nucleates as a liquid meniscus preferentially in the cavity between the particle and the substrate; no unattached droplet is found on the substrate over a region of about 100 μm × 100 μm. Initially, the nucleated droplet is not well resolved, since the contact line is obscured by the particle itself. Later, the growing droplet radius exceeds the particle radius and could be resolved.

**Figure 5:**
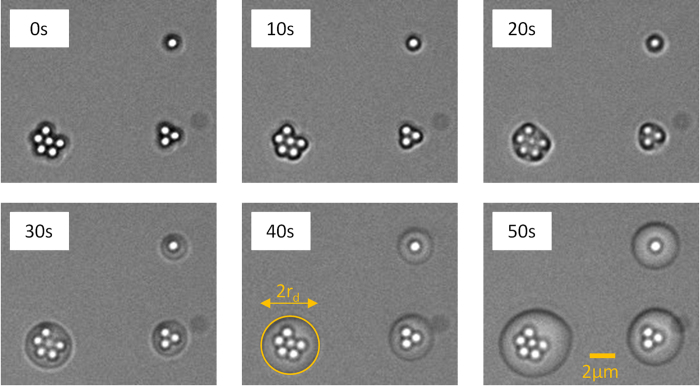
Time resolved images of droplet growth on a single particle, three-particle and six-particle clusters in ten-second increments. The apparent radius is determined by close-fitting the droplet outline to a circle, an example illustrated in the image for t = 40 s.

Figure 5 shows images of droplet growth on a single particle, three-particle and six-particle clusters in ten-second increments from initial time t = 0 s, when the first flicker of droplet contact line is observed at the edge of the particles. During early times, the nascent droplet perimeter follows the peripheral outline of the cluster; the droplet outline becomes circular by t = 30 s onwards. The apparent radius is determined by close-fitting the droplet outline to a circle, an example illustrated in the image for t = 40 s. Based on the even illumination and lack of optical distortion throughout the experiment, it appears likely that the droplet has not completely wetted the particle up to t = 50 s.

**Figure 6:**
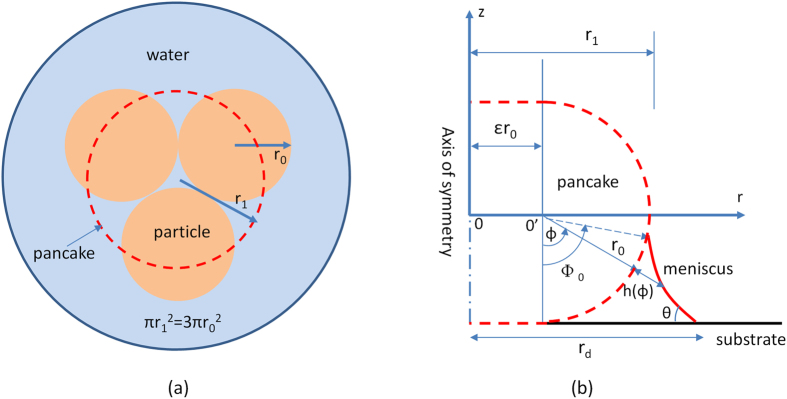
Schematic diagram of a three-particle cluster approximated by a pancake model shown here as a dashed circle on (**a**) top-down view, and as a solid body on (**b**) side view.

For a tightly grouped particle cluster, we approximate the collective geometry as a single pancake model structure as shown in Fig. 6a (for a three particle cluster). The pancake model consists of a cylindrical core, conjoined and wrapped around the sides by a revolved semi-circle of radius r_0_, which is equivalent to the radius of a single particle. The apparent centerline radius of the pancake r_1_ is set by equating the centerline pancake area πr_1_^2^ and the sum of all actual particle areas nπr_0_^2^, where n is the number of particles in a cluster (Fig. 6b). This geometric reshape, while simplistic, retains features of dimensional scaling and morphological consistency at the edges.

For the pancake model, we express the Young-Laplace equation in Eq. (1) as


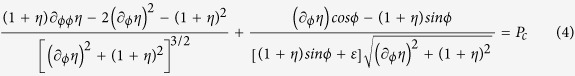


where ε scales the interior cylindrical core as (*r*_*1*_/*r*_0_) − 1. The numerical solution follows the same scheme as indicated earlier for the case of a single particle.

**Figure 7:**
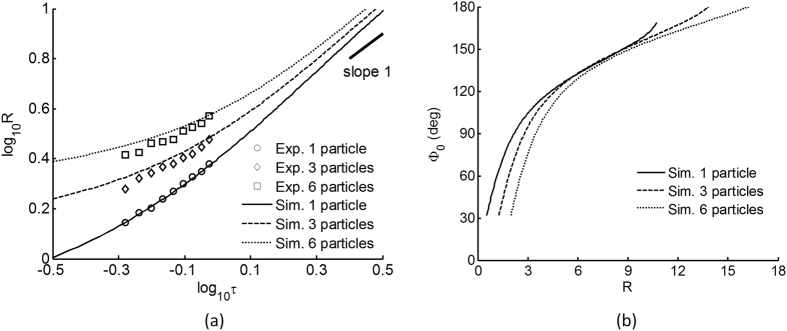
(**a**) Logarithmic time-plot of non-dimensional droplet radius for the cases of single particle, 3-particle and 6-particle clusters for both experimental (markers) and simulated (lines) data. Condensation rate is 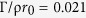 s^−1^ and the contact angle is 22°. Eye-guide of slope 1 refers to an idealized growth rate of a particle-free droplet. (**b**) Plot of droplet aspect ratio represented by wetting edge Φ_0_ and the non-dimensional radius R.

The droplet is simulated using a contact angle of 22° (experimentally determined) and an initial wetting edge Φ_0_ of 30°. The resulting droplet wets the substrate to a radius of approximately R so the contact line is at the threshold of experimental visibility. The droplet grows at rate determined by the condensation mass flux Γ. As shown in Fig. 7a, we fit the simulated droplet growth rate to experimental data (obtained from Fig. 5), using a condensation rate constant 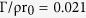 s^−1^. For a water droplet of density ρ = 1000 kg/m^3^ and particle radius r_0_ = 1 μm, the dimensional mass flux Γ = 21 mg/m^2^s.

Here, we make the case that a good trend fit is not assured simply by tuning a single free parameter. In particular, we find that the direct condensation mechanism, which predicts a linear growth rate^23^,^24^,^25^, caters sufficiently to the fit on a constant flux. Other condensation models, for example substrate monomer diffusion and limited directed condensation, predict a growth power law between one-third^8^,^9^,^10^,^11^ to one-half^20^, but they do not fit the experimental data trend reasonably. In addition, we found that the apparent initial deviation from linear growth of slope one occurs due to the geometric and wetting effects of the particle. This observed deviation diminishes as the droplet size becomes large compared to the particle, such that the growth behavior approaches that of homogeneous condensation.

As further validation, we simulate the cases for three and six particle clusters using the pancake model as described earlier. Using the same condensation rate constant 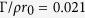 s^−1^ as for the single particle case, the simulated growth curves are found to be in good agreement with the actual experimental data, without any fitting (Fig. 7a). This result indicates that both the pancake geometric approximation and the assumed condensation flux are reasonable assumptions for our simulations.

Further, we compare the respective late time growth rates against the theoretical linear growth rate of a particle-free droplet due to a constant condensation flux (see eye-guide of slope 1). Interestingly, we note that the growth curve for the particle clusters (pancake model) finds better agreement with the indicated slope than for the single particle. To clarify this, we plot the aspect ratio based on the wetting edge Φ_0_ and non-dimensional droplet radius R, as shown in Fig. 7b. This plot essentially describes the ratio of wetting on the particle against the substrate, and indeed, the droplet wets the particle clusters faster than the single particle case, so that further droplet growth tends to wet the substrate instead of the particle. This results in a growth rate similar to that of a homogeneous droplet (slope 1).

For the case of a single particle, we note that the out-of-plane curvature term rapidly becomes singular as the droplet approach complete wetting, leading to a wetting-volume transition as previously discussed (Fig. 2). Conversely, for the case of particle cluster (pancake model), this problem is alleviated, for even as wetting edge Φ_0_ approaches 180°, the out-of-plane radius of curvature tends to 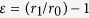, and not zero. Therefore, the wetting-volume transition is not significant for the particle clusters. This is particularly evident for the 6-particle case as depicted in Fig. 7b for larger values of Φ_0_.

## Discussion

We had presented herein an analysis on the condensation of water on micron-sized particle or cluster of particles on a substrate, specifically on drop growth and wetting behavior. Notably, alternative thermodynamic wetting states are accessible as the growing droplet approach complete wetting of the particle. Partially wetting droplet profiles become less favorable as the out-of-plane curvatures approach singularity. This, we supported with calculations of Gibbs energy subjected to contact angle and wetting edge, and obtained a transition curve where a jump from partial to complete wetting may occur.

In addition, we simulated droplet growth under experimental conditions, and found a good agreement with experimental results using a single fitting condensation mass flux constant. The analysis was extended to particle clusters using a simple geometric approximation in the form of a pancake, and our results are also consistent with experimental observations.

With that, we ask the question of how droplet profiles evolve during condensation growth, but constrained by geometric and wetting considerations. Here, we present a case where the wetting behavior of either the particle or the substrates is competitive and unbalanced, even with the same contact angles. This result highlights a gap in our understanding of condensation and surface wettability at small scales. The present work could be extended to problems involving capillary adhesion between wetted particles and substrates, or wetted fine particulates and granules commonly used in chemical industries.

## Additional Information

**How to cite this article**: Quang, T. S. B. *et al*. Growth and wetting of water droplet condensed between micron-sized particles and substrate. *Sci. Rep.* **6**, 30989; doi: 10.1038/srep30989 (2016).
